# The Late Samuel Tuke, Esq., of York

**Published:** 1858-01-01

**Authors:** 


### THE LATE SAMUEL TUKE, ESQ., OF YORK.

(*Extracted from the* "*York Herald*" *of October* 17, 1857.)

Our obituary of this day will recal a name fraught with no common interest to many of our citizens. Recollections worthy of being retraced will be aroused in some minds—a sympathetic feeling in many—a respectful recognition of departed worth, perhaps we may say, in all.

It is one of the most interesting features of the social framework of Britain, that while it recognises the distinctions of feudal rank, and records the exit of each worthy head of a time-honoured house, as in some sort the property of the nation, not the less through the various gradations of the scale does it appreciate the successful citizen, the independent yeoman, or even the lowly mechanic, if such an one, filling worthily his station, or rising to a higher sphere, has left to his successors incentives to the like honourable course—"footprints on the sands of time."

Of the burgher or citizen class, was the immediate family of Samuel Tuke. The name of Tuke, early scattered in Nottinghamshire and South Yorkshire, appears in the seventeenth century in the city of York, where the ancestor of the subject of this sketch, having embraced the principles of the Quakers, suffered imprisonment in consequence, in "Ouse Bridge Prison," in the year 1660.

Samuel Tuke was the eldest grandson of William Tuke, who died in 1822, at the patriarchal age of 90, and whose name is so well known as the founder of the Friends' Retreat, near York, in 1792, and as the originator in this country of those principles in the treatment of insanity, which, in their progress, have so much contributed to the alleviation of human suffering.

William Tuke's eldest son, Henry Tuke, died at the comparatively early age of 58, not less honoured and beloved than his father.

Samuel Tuke, the only son of Henry who lived to maturity, was born 31st July, 1784. He early co-operated with his grandfather and father in their philanthropic labours. To the subject of insanity especially, as is well known, he devoted a large portion of his time, and in the course of his life was the author of several works which are well known on the Continent and in America, as well as in this country. His "Description of the Retreat" was published in 1813, and led to very remarkable consequences—consequences which the author himself had never ventured to anticipate. Steadily did he pursue his labours in the great work of bettering the condition of the insane, not only by his writings, but by the unremitting attention which he paid to the welfare of the Retreat, of which he was the treasurer for thirty years. Not inaptly has he been called "the Friend of the Insane."

In 1840, he edited the work of a German physician, Dr. Jacobi; in the introduction to which he fully expresses his views in regard to the provision for the insane, and their moral management, with many practical directions regarding the construction of asylums.

But to many of the readers of this memoir it is as the public man and the active citizen that Samuel Tuke will be chiefly remembered. To some, as the man of warm, deep, and abiding sympathies, in private life; to not a few by the earnestness, the deeply devotional spirit, the catholicity of feeling, yet lofty standard of Christian obligation, which marked his religious character.

He was never a party man. His mind was simply incapable of being so moulded. Every line of action which he adopted, however much it might provoke hostility in those who honestly took a different view, was simply the result of some great principle, firmly grasped and rigidly carried out. Thus, he early supported the concession of political privileges to the Roman Catholics, when a very different view might have been expected from association and training. Yet his mind was essentially conservative, in the sense of a deep feeling of the *venerable*—intense in proportion to the moral worth associated with it. Equally strong was his love of social order—his idea of government as the embodiment of a governing moral force.

The period of his life comprised events of no ordinary political interest and importance. The contested election for the county of York in 1807; the abolition of the Slave Trade, and the struggle for the extinction of the system of Slavery; the Reform Bill of 1832, and the carrying out of its spirit and principles, may be mentioned as subjects in which he felt and manifested a warm interest.

There was, we believe, only one occasion on which he appeared before the public in any sense as a political partisan. In the year 1833, on the election of the Hon. Thomas Dundas to fill a vacancy in the representation of the city of York, having been himself solicited to stand, he gave the full weight of his eloquence in support of that gentleman. This was very much prompted by an ardent wish to carry out those principles to which we have already alluded, and which, in his mind, were inseparably connected with the idea of a true Reform in the Representation.

It was, however, in support of the claims of the British and Foreign Bible Society—in Anti-slavery efforts—the cause of Scriptural education of the poor—and various movements of a philanthropic or religious character, that his influence and his voice were most frequently exerted.

We might, were it needful, enumerate the various charitable institutions of the city as partakers of his pecuniary or active personal assistance. Judicious Benefit Societies for the Working Classes—Sanitary Reform—his active and unremitting exertions when guardian of the poor—will naturally be suggested to the minds of those who may have watched his public life, or shared his labours. In this last-named capacity, his sympathy with suffering and intense aversion to anything bordering upon oppression, were obvious features of his character.

Samuel Tuke's mind was a rare combination, comprising a sound judgment with no small measure of more shining qualities. To a vigorous and perceptive intellect, he united a vivid imagination, and a strong sense of the beautiful. He was therefore a man of taste—rigidly correct *taste*. His eloquence, though somewhat unequal, was of a striking and often lofty character. There was a masterly comprehension of an idea—forcible, clear, and well-enunciated expression. On certain occasions the clear summing-up of conflicting arguments, and the delivery of a lucid judgment with calm precision, yet always with a certain warmth of feeling, elicited a display of mental power not easily forgotten.

The preceding slight outlines will be readily filled up by those who knew the man, —not less readily when we allude to him as the kind neighbour, the unwearied benefactor to the poor, or the fellow-citizen, sharing in

> "—————— the talk
> Man holds with week-day man in the hourly walk
> Of the mind's business."

We must not omit to say that Samuel Tuke was a *man of business*. He was long the head of a prosperous firm, succeeding to the concern founded by his grandfather, now about a century ago. The unfailing energy and varied talents of a mind whose home was in far higher pursuits, precluding him from being less than the presiding mind of the whole,—these were best understood by persons brought into intimate association with him in this character.

The sanctuary of the domestic hearth with such a mind was indeed a sanctuary; and only the large and happy family who revered him as a parent can fully understand the associations which this allusion may call forth. After eighteen years of married life, he was called upon to endure the severest trial which human affection can undergo. But the man, or rather the Christian, though "cast down was not destroyed;" and soon was he again active in the field of duty, with energies only deepened by the shade of sorrow. His active intellect hardly seemed to admit of repose. It had been well, indeed, if such a mind had more of the disposition to relax. Playfulness was not an element in his character, which was naturally stern, but not the less was there the flow of natural wit, and at times a chastened humour more delightful still. His religious character may be touched upon—briefly, because of the sacredness of the subject; confidently, because it was the substratum of his moral being—at once the spring and the regulator of his energies. We would fain appeal to those who, alas! are no more household names in our city—the names, well recognised in their day, of William Gray, John Graham, Anthony Thorp, Thomas Wemyss—as members of a vanished circle (as we can confidently to not a few still living), who would instantly appreciate the soundness and stability of his Christian character.

As a member of the religious Society of Friends, by conviction as well as by birth, he was, as in everything else, the active exemplar of the principles he adopted. He carried them out for himself, even in their remoter bearings; but surely we need not again say that Samuel Tuke belonged less to a sect than to the universal Christian Church.

